# An outbreak of bovine trypanosomiasis in the Blue Nile State, Sudan

**DOI:** 10.1186/1756-3305-4-74

**Published:** 2011-05-13

**Authors:** Bashir Salim, Mohammed A Bakheit, Sir Elkhatim Salih, Joseph Kamau, Ichiro Nakamura, Ryo Nakao, Chihiro Sugimoto

**Affiliations:** 1 Department of Collaboration and Education, Research Center for Zoonosis Control, Hokkaido University, Sapporo 001-0020, Japan; 2 Department of Parasitology, Faculty of Veterinary Medicine, University of Khartoum, 13314 Khartoum-North, Sudan; 3 Veterinary Infection Biology and Immunology, Research Center Borstel, D-23845 Borstel, Germany; 4 Damazin Veterinary Laboratory, Blue Nile State, Federal Ministry of Animal Resource and Fisheries, Sudan

## Abstract

**Background:**

In this paper, we report an outbreak of bovine trypanosomiasis in Kurmuk District, Blue Nile State, Sudan that involved an infection with four *Trypanosoma *species in cattle. The outbreak occurred in June 2010 when indigenous cattle, mainly Kenana and Fulani breed types, crossed the national Sudanese border to Ethiopia and returned. A veterinarian was notified of massive deaths in the cattle populations that recently came from Ethiopia. All animals involved in the outbreak were from the nomadic Fulani group and resident local cattle were not infected and no death has been reported among them. A total of 210 blood samples were collected from the ear vein of cattle. A few samples were also collected from other domestic animals species. Parasitological examinations including hematocrit centrifugation techniques (HCT) and Giemsa-stained thin blood films were carried out. ITS1-PCR, which provides a multi-species-specific diagnosis in a single PCR, was performed.

**Findings:**

Parasitological examinations revealed that 43% (91/210) of the affected cattle population was infected with two morphologically distinct trypanosomes. Seventy animals (33.3%) were infected with *T. vivax *and twenty one (10%) with *T. congolense*. In contrast, ITS1-PCR was able to identify four *Trypanosoma *species namely *T. vivax, T. congolense, T. simiae *and *T. brucei *in 56.7% (80/141). *T. brucei *showed the highest prevalence of 36.9% (52/141) and the lowest 19% (27/141) was displayed by *T. congolense*. Furthermore, and because ITS1-PCR could not differentiate between *T. brucei *subspecies, serum resistance-associated (SRA) gene based PCR was used to detect the human *T. brucei rhodesiense *in *T. brucei *positive samples. None of the samples was shown positive for *T. b. rhodesiense*. The identity of the 400 bp PCR product originating from *T. simiae*, was further confirmed by sequencing and subsequent phylogenetic analysis.

**Conclusions:**

The outbreak of bovine trypanosomiasis occurred in the Blue Nile State was caused by mixed infection of two or more *Trypanosoma *species and the conventional parasitological examinations were not reliable in identifying all the species of *Trypanosoma *involved in the outbreak. It is difficult to determine the cause of the disease for the reason that the current enzootic situation in the resident cattle in the region is poorly understood. The study concluded that there are at least four species of trypanosomes that caused this outbreak in the Blue Nile State. The presence of mixed infections might have exacerbated the severity of the disease. It is hypothesized that variant parasite type(s) might have been introduced to Sudanese cattle when they crossed to Ethiopia, a tsetse belt region.

## Background

Cattle population in the Blue Nile State, Sudan, is estimated to be 1,995,024 heads (Annual Report of Ministry of Animal Resources and Fisheries, Sudan, 2009). Generally, data on bovine trypanosomiasis and the vector *Glossina *spp. (tsetse fly) present in the Blue Nile State and neighboring states is scarce and only one report showed the existence of *Glossina fuscipes fuscipes *and *G. morsitans submorsitans *in Kurmuk District [1].

This study has been carried out to determine the *Trypanosoma *species which were incriminated in an outbreak of bovine trypanosomiasis in Kurmuk District, the Blue Nile State. It is evident that disease risk depends primarily on the density of the vector and trypanosome infection rate. The affected animals were closer to Ethiopian tsetse-belt where nomads cross in with their animals/cattle in search of pasture and water. The tsetse flies in Ethiopia are confined to the southern and western regions between longitude 33°and 38°E and latitude 5°and 12°N [2]. This region overlaps with the area where two *Glossina *species were reported in the Blue Nile State, Sudan [1], though there was no report showing the tsetse belt is reaching the Blue Nile State in the Sudan.

### Sample collection and parasitological examination

To preserve owners' confidentiality and to adhere to the International Ethical Guidelines for Biomedical Research involving animal subjects, no owner names were recorded within any database or as part of the data collection process. The owners of the sampled cattle provided consent to have their animals included in the study. Research on samples was conducted adhering to guidelines of the Institutional Animal Care and Use Committee of the Graduate School of Veterinary Medicine, Hokkaido University. The study protocol also followed the general guidelines for sampling domestic animals provided by the Faculty of Veterinary Medicine, University of Khartoum, Sudan.

**Figure 1:**
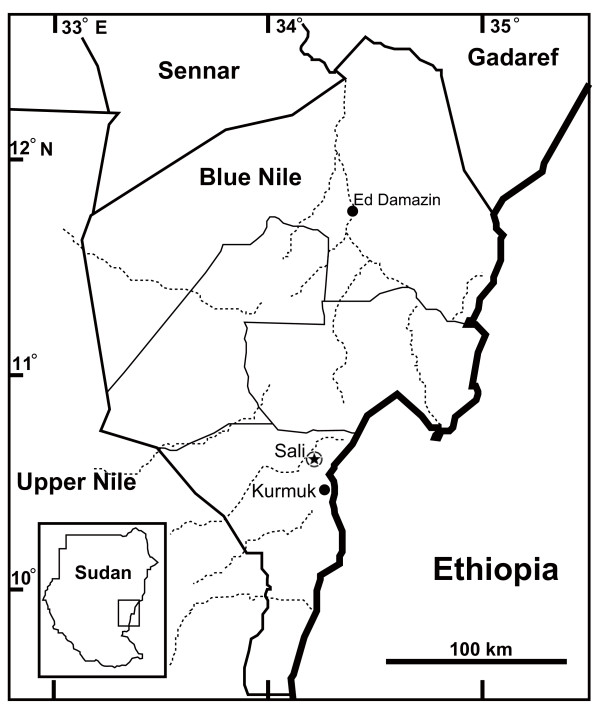
**Map of the Blue Nile State**. The outbreak location is shown in black star.

The outbreak occurred in June 2010 (Rainy season). On the 13^th ^of June 2010, a total number of 210 blood samples was collected from affected cattle in Sali village at Kurmuk District (10°78'N 34°18'E) in the Blue Nile State, Sudan (Figure 1). Three millilitres of blood were collected from the ear vein in heparinized vacutainer tubes (Becton Dickinson, France). A few more samples were similarly collected from donkeys, camels and sheep in the village (Table 1). Parasitological examinations were carried out in the regional laboratory using HCT according to [3]. Subsequently, 10% Giemsa-stained thin blood smears were prepared and examined under oil immersion field to identify trypanosomes species based on their morphological structures. For PCR, 141 samples were spotted onto FTA^® ^elute cards (Whatman, Inc, UK), which included the 91 parasitologically positive samples and additional 50 randomly selected negative samples.

**Table 1:** Parasitological examinations of cattle and other domestic animals samples during an outbreak of cattle trypanosomiasis in the Blue Nile State, Sudan

Animal	No. samples	HCT (%)	No. positive samples by thin film	*Trypanosoma *spp. identified (number, per cent)
Cattle	210	43	91	*T. vivax *(70, 33.3%) *T. congolense *(21, 10%)
Donkeys	8	37.5	3	*T. vivax *(3, 37.5%)
Camels	2	100	2	*T. evansi *(2, 100%)
Sheep	3	66.7	2	*T. vivax *(2, 66.7%)

### DNA extraction

Genomic DNA was extracted from six punches in each FTA card to ensure correct estimation of prevalence as described elsewhere [4-6]. Briefly, blood samples collected on FTA cards were dried thoroughly at room temperature. Using a sterile punch, each FTA card was punched out at 6 different positions each was 3 mm size. These were placed into a sterile microcentrifuge tube and rinsed 3 times each in 750 μL of deionized water by vortexing for 5 seconds and discarding of water. DNA was eluted using a buffer that contained 90 μL deionized water plus 10 μL 10X ThermoPol Reaction Buffer (Biolabs, Inc, England). Elution was performed by heating the sample at 95°C for 30 minutes using a heat block. Eluted DNA concentration ranged between 100 - 250 ng/μL. DNA was stored at -20°C until used.

### PCR

Extracted DNA was subjected to a PCR test, which amplifies the ITS1 region of the rDNA gene of all African trypanosomes by using ITS1 CF/BR CF: 5'-CCGGAAGTTCACCGATATTG-3', BR: 5'-TGCTGCGTTCTTCAACGAA-3' [7]. The 700, 480, 400, and 250 bp of PCR products corresponding to *T. congolense *(savannah), *T. brucei *subspecies and *T. evansi, T. simiae *and *T. vivax *respectively, were amplified using GoTaq^® ^Colorless Master Mix, 2X (Promega Co., USA) in a 10 μl total volume. Each reaction included 5 μl GoTaq^® ^Colorless Master Mix, 10 mM of each primer, 1 μl RNase-free water and 2 μl (~100 ng/μl) extracted DNA. To detect *T. brucei rhodesiense*, the following primer set was used FP: 5'-ATA GTG ACA AGA TGC GTA CTC AAC GC-3' and RP: 5'-AAT GTG TTC GAG TAC TTC GGT CAC GCT-3'. This primer set targets the serum resistance-associated gene (SRA) and amplifies a 284-bp fragment [8]. Thermocycling profile started with an initial hold for 2 min at 95°C, followed by 35 cycles of 95°C for 30 sec, 58°C for 30 sec. and 72°C for 1 min. A final extension step of 5 min at 72°C ended the program. PCR products were electrophoresed in 2% agarose Zebra (BioTools, Inc, Japan) in TAE buffer and stained using GelRed dye (Biotium, Inc., USA) before being visualized under UV light.

As *T. simiae *is generally assumed to be a pig pathogen, and in order to confirm the identity of the 400 bp PCR product as originating from *T. simiae*, ITS1-PCR products obtained for three randomly selected *T. simiae*-positive samples were cleaned using Wizard^® ^SV Gel and PCR Clean-Up System (Promega, WI, USA) and sequenced using forward and reverse primers according to the protocol of the Dye Terminator V.3.1 cycle sequencing kit (Applied Biosystem, Japan). The products were subsequently ethanol/EDTA/sodium acetate precipitated prior to loading to ABI Prism 3730 Genetic analyzer. Sequence chromatograms were edited and analyzed using the software Finch TV Version 1.4.0 (Geospiza, Inc., USA) and ApE http://www.biology.utah.edu/jorgensen/wayned/ape/. Sequences of (~340 bp) were blasted in NCBI for sequence similarity and phylogenetic analysis to depict the relationship with other *Trypanosoma *spp. Sequences were deposited in the Genbank under the accession numbers [Genbank:AB625444, AB625445 and AB625444].

## Results and discussion

The results of HCT and Giemsa-stained blood films revealed that 43% (91/210) of the cattle were infected with two morphologically distinct trypanosomes. Seventy (33.3%) were infected with *T. vivax *and twenty one (10%) with *T. congolense *(Table 1). The ITS1 PCR identified four *Trypanosoma *spp (Table 2). The mortality estimated for 4 herds (150 - 300 heads/herd) visited ranged between 20 - 90%.

**Table 2:** Prevalence of tsetse transmitted trypanosomes as revealed by ITS1 PCR detection method in cattle samples during an outbreak of cattle trypanosomiasis in the Blue Nile State, Sudan

*Trypanosoma *spp. infection	Prevalence (%)
*Tc + Tbb + Ts + Tv*	1 (0.7)
*Tc + Tbb + Ts*	5 (3.5)
*Tc + Tbb + Tv*	1 (0.7)
*Tbb +Ts + Tv*	7 (5.0)
*Ts + Tv + Tc*	1 (0.7)
*Tc + Tbb*	6 (4.3)
*Tc +Ts*	6 (4.3)
*Tc + Tv*	5 (3.5)
*Tb + Ts*	5 (3.5)
*Tb + Tv*	5 (3.5)
*Ts+ Tv*	0
*Tc*	2 (1.4)
*Tb*	**22 (15.6)**
*Ts*	6 (4.3)
*Tv*	8 (5.7)
**Overall prevalence of each species**	
*Tbb*	**36.9% **(52/141)
*Ts*	22% (31/141)
*Tv*	20% (28/141)
*Tc*	19% (27/141)
**Overall prevalence of all species**	**56.7% (80/141)**

Tc, Tbb, Ts and Tv represent *T. congolense, T. b. brucei, T. simiae *and *T. vivax *respectively.

**Figure 2:**
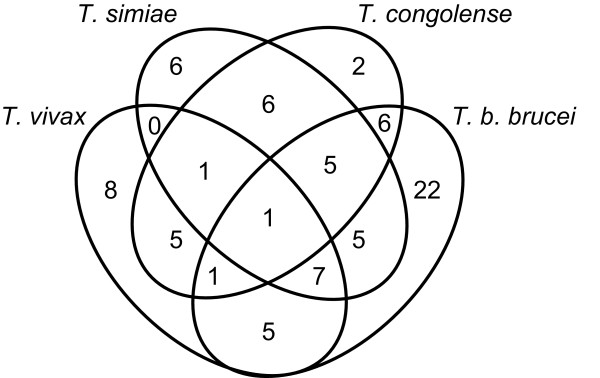
**Single and mixed infections of four *Trypanosoma *spp. detected in cattle by ITS1-PCR in the Blue Nile State Sudan**.

**Figure 3:**
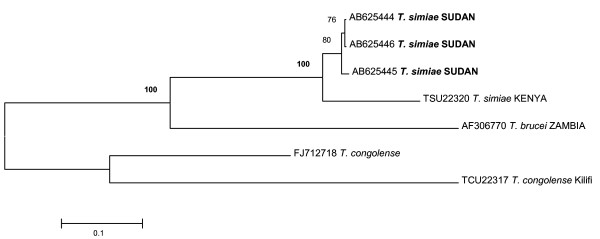
**Rooted phylogenetic tree showing the relationship of the *T. simiae *identified in this study with reference sequences of *T. simiae, T congoloense *and *T. brucei***. The relationship was determined using the ITS1 of rRNA gene sequences by neighbor joining with 1,000 bootstrap. *T. simiae *samples identified in this study were depicted in bold letters. Trypanosomes sequences from GenBank were shown both by their accession numbers and parasites names). Scale bar used was nucleotide substitutions per position

The results obtained by ITS1-PCR indicated that cattle were infected with four *Trypanosoma *spp. and most of the infection patterns were of mixed nature. We could detect simultaneous infection with 3 and even 4 *Trypanosoma *spp in a single animal (Figure 2). A total number of 80 cattle were infected, giving an overall prevalence of 56.7% (80/141) in these samples. *T. b. brucei *showed the highest prevalence of 36.9% (52/141), followed by *T. simiae*, 22% (31/141), *T. vivax*, 20% (28/141) and *T. congolense*, 19% (27/141) (Table 2). It is worth noting that the PCR overall prevalence shown in this study does not constitute an accurate prevalence of trypanosomiasis in the affected population, as the majority of samples selected for PCR were known parasitologically positive. Nonetheless it is clearly reflected here that ITS1-PCR has better specificity for detection of trypanosomes. The 400 bp fragments obtained from three randomly selected *T. simiae*-positive samples were confirmed to be *T. simiae *by direct sequencing of the ITS1 region. The *T. simiae *detected in the in this study showed similarity to *T. simiae *from Kenya (accession no. TSU22320). Phylogenetic relationship of the *T. simiae *identified in this study to reference sequences of *T. simiae, T congolense *and *T. brucei *is given in (Figure 3).

There are three known genetically distinct types of *T. congolense *(savannah, forest and kilifi). Only the savannah type of *T. congolense*, (PCR fragment size of 700 bp) was detected, while the other two, *T. congolense *kilifi (620 bp) and *T. congolense *forest (710 bp), were not. *T. congolense *savannah is reported to be of extreme virulence [9].

The conventional parasitological methods were not able to detect and/or discriminate between *T. simiae *and *T. b. brucei *though the latter had the highest prevalence when we used ITS1 PCR detection methods. The low microscopic detection rate might be due to chronic enzootic nature of *T. b. brucei*, in which the level of parasitaemia is usually below the level of detection offered by the conventional methods. On the other hand, *T. simiae *might be misdiagnosed as *T. congolense *as the two parasites are known to be morphologically similar [10].

This study represents a first report on the presence of *T. simiae *in Sudan. It is worth mentioning that large numbers of wild boars (*Sus scrofa*) do exist in the Blue Nile State. The presence of wild boars, which graze in vicinity of domestic cattle in the region, could be linked to the presence of *T. simiae *detected in cattle during this study. It remains to be confirmed whether this animal could indeed act as a natural reservoir to this species of *Trypanosoma*.

Generic ITS1-PCR was developed by [11] for trypanosome detection. This method was modified to increase the sensitivity of the primers to *T. vivax*, an important cattle trypanosome [7,12]. In addition, [13,14] have further refined the test to increase the sensitivity and specificity in detecting trypanosomes. The method was shown to be a powerful diagnostic technique for detecting and identifying trypanosomes quickly, accurately and cheaply. In our study, ITS1-PCR detected lesser number of positives (80) compared to the parasitological methods (91) in the same samples. Collected blood was kept in the fridge before it was spotted in the FTA cards, thus might become subjected to DNA degradation and subsequently underestimated the accurate sensitivity of the ITS1-PCR.

An additional species-specific PCR was sometimes needed when the ITS1-PCR products of some species were too similar or same in size to distinguish, for example *T. brucei *subspecies and *T. evansi *or *T. simiae *and *T. simiae Tsavo *[14]. Because ITS1-PCR could not differentiate between *T. brucei *subspecies, we used the serum resistance-associated (SRA) gene based PCR to detect the human *T. brucei rhodesiense *in *T. brucei *positive samples. However, none of the samples was shown positive for this species.

From the result showed in Table 1, other domestic livestock (donkeys, camels and sheep) were infected by trypanosomes based on microscopical examination. Although local resident cattle were not sampled during this study, the presence of infection in other resident animal species should imply that some if not all trypanosome species involved in this outbreak are also present in resident cattle in the region. Urgent screening of possible trypanosome species in resident cattle in the region using ITS1-PCR is required for understanding of the enzootic nature of the disease.

The study concluded that there are at least four species of trypanosomes that caused an outbreak of trypanosomiasis in cattle in the Blue Nile State. The presence of mixed infection might have exacerbated the severity of the disease. Though we hypothesized that variant parasite type(s) might have been introduced to Sudanese cattle when they cross into Ethiopia, a tsetse belt region. We conclude that future research should focus towards a better understanding of the picture of parasite endemicity for better control measures. This plan should also include surveillance for the possible *Glossina *and other biting fly species existing in the region.

## Competing interests

The authors declare that they have no competing interests.

## Authors' contributions

BS carried out the molecular genetic analyses, participated in the data analysis, did the field collection, participated in the statistical analysis and drafted the manuscript. MAB was involved in field collection and helped to draft the manuscript. SS performed parasitological examination and involved in field collection. JK, IN and NR participated to draft the manuscript. CS helped to conceive the study, participated in its design, assisted in obtaining funding and helped to draft the manuscript. All authors read and approved the final manuscript.
